# Multi-Pathway Antifungal Mechanism of Marine-Derived *Bacillus* sp. BAF143 Against *Aspergillus flavus*

**DOI:** 10.3390/md24090325

**Published:** 2026-09-17

**Authors:** Xiaoyun Ou, Youzhi Li, Shaojie Wang, Qiaozhen Wang, Shushi Huang, Futian Yu, Yuening Luo, Min Liang, Ling Yang, Lixia Pan, Xiaochun Wang, Dengfeng Yang

**Affiliations:** 1 State Key Laboratory of Non-Food Biomass Energy Technology, Guangxi Key Laboratory of Marine Natural Products and Combinatorial Biosynthesis Chemistry, Guangxi Academy of Marine Sciences, Guangxi Academy of Sciences, Nanning 530007, China; 13005900227@163.com (X.O.);; 2 College of Life Science and Technology, Guangxi University, Nanning 530004, China; 3 Department of Traditional Chinese Materia Medica, Shenyang Pharmaceutical University, Shenyang 110016, China

**Keywords:** *Bacillus* sp. BAF 143, *Aspergillus flavus*, antifungal mechanism, transcriptomics, biocontrol

## Abstract

*Aspergillus flavus* is a major fungal pathogen that causes postharvest spoilage and carcinogenic aflatoxin contamination in agricultural commodities, necessitating the development of novel biocontrol strategies. In this study, a bacterial strain of *Bacillus* sp. BAF143, isolated from a Martin medium plate used for marine fungal cultivation, exhibited potent antifungal activity against *A. flavus*. The crude extract of BAF143 demonstrated strong inhibition of mycelial growth (62.36 ± 1.00%) and spore germination (90.10 ± 0.21%). Transcriptomic analysis revealed a distinctive response pattern: genes involved in ribosome biogenesis, oxidative phosphorylation, ergosterol biosynthesis, cell cycle, DNA replication, and energy metabolism were significantly upregulated, whereas cell wall synthesis and MAPK signaling pathway genes were downregulated. This paradoxical transcriptional landscape indicates that *A. flavus* mounted a desperate compensatory response to counteract cellular damage, which was ultimately overwhelmed by excessive reactive oxygen species accumulation, lipid peroxidation, and mitochondrial dysfunction. Physiological assays confirmed membrane integrity loss, mitochondrial membrane potential collapse, and DNA fragmentation, leading to apoptosis-like cell death. On peanuts, the crude extract achieved a 96.18% reduction in *A. flavus* spore count after 21 days with sustained protection. These findings demonstrate that the BAF143 crude extract exerts a multi-pathway antifungal mechanism, positioning *Bacillus* sp. BAF143 as a promising biocontrol agent for mitigating *A. flavus* contamination and aflatoxin risks in postharvest agricultural products.

## 1. Introduction

*Aspergillus flavus* is a ubiquitous saprophytic fungus that can infect various food crops, and poses a significant threat to global food security and public health. As a predominant contaminant of staple crops such as maize, peanuts, nuts, soybeans and cottonseeds, especially when stored in warm and humid conditions [1], *A. flavus* results in substantial economic losses annually and exposes millions of populations in developing regions to high-risk dietary toxins [2,3,4]. It not only causes severe post-harvest spoilage but also produces secondary metabolite aflatoxin B1 (AFB1). AFB1 was classified as a Group 1 carcinogen by The International Agency for Research on Cancer (IARC) of the World Health Organization (WHO) [5]. It damages liver tissues and leads to liver cancer and even death in humans and animals. Therefore, the prevention and control of *A. flavus* constitute a matter of considerable urgency.

Currently, various strategies are used to control the growth of *A. flavus* and aflatoxin production, including chemical fungicides, physical treatments, and biological control agents. However, more and more concerns regarding chemical residues, environmental pollution, and fungal resistance have prompted a shift towards safer, eco-friendly alternatives. Biological control using antagonistic microorganisms or plants and their bioactive metabolites and enzymes to manage the contamination of *A. flavus* and aflatoxins [6,7] is more effective and environmentally friendly, and has emerged as a promising strategy. Plant-derived bioactive compounds include essential oils [8,9,10], phenolic compounds [11,12], aromatic aldehyde [13,14], and antimicrobial peptides [15]. Various microorganisms and their bioactive metabolites have been identified as effective inhibitors of *A. flavus* mycelial growth and aflatoxin production. These include *Bacillus* spp., which can produce methylmalonic acid, surfactin, iturin A, fengycin, bacillomycin, and peptides (L-Asp-L-Orn, L-Asp-L-Asn and L-Asp-L-Asp-L-Asn) [6,16,17,18]. *Streptomyces* spp. produces VOCs like hexanal, 1-octen-3-ol, and 2-pentylfuran [19]. Among fungi, several non-aflatoxigenic *Aspergillus* species, such as *A. oryzae* and *A. fumigatus* [20,21], as well as yeasts including *Wickerhamomyces subpelliculosus* and *Saccharomyces cerevisiae*, which produce ethyl acetate and benzothiazole [22], and mushrooms [23,24,25], have been reported to exhibit antifungal activity against *A. flavus*.

Among bacterial antagonists, *Bacillus* spp. are the most extensive and prominent, and have long been recognized as Generally Recognized as Safe (GRAS) biocontrol microorganisms. Numerous studies have documented the ability of *Bacillus* species and their bioactive secondary metabolites to inhibit *A. flavus* growth and aflatoxin productions. Many *Bacillus* species, particularly *B. albus* [26], *B. amyloliquefaciens* [17,27], *B. megaterium* [18], *B. siamensis* [28], *B. subtilis* [29], *B. velezensis* [6,17,30], etc., have been reported to exhibit potent antagonistic activity against *A. flavus* through the production of diverse secondary metabolites. Among these, lipopeptides such as surfactin, iturin A, fengycin, and bacillomycin are the most well characterized [6,16,17], functioning primarily by disrupting fungal cell membrane integrity and permeability, leading to leakage of cellular contents and ultimately cell death [31,32]. Beyond lipopeptides, *Bacillus* spp. also secrete antimicrobial peptides (e.g., D_1_O, D_1_N, and D_2_N) [18], hydrolytic enzymes (including glucanases and chitinases) [33], and volatile organic compounds [34,35], all of which synergistically contribute to fungal growth inhibition. Notably, certain *Bacillus* metabolites have been shown not only to suppress mycelial proliferation but also to downregulate the expression of key genes within the aflatoxin biosynthetic gene cluster (*aflR*, *aflS*, and structural genes such as *aflD* and *aflO*), thereby drastically reducing aflatoxin B1 production [6]. Despite these promising findings, the comprehensive antifungal mechanisms of *Bacillus* metabolites, particularly their integrated effects on cellular ultrastructure, oxidative stress homeostasis, genomic integrity, and global transcriptional reprogramming, remain insufficiently elucidated. Therefore, a systematic investigation integrating physiological and transcriptomic approaches is essential to fully unravel the multifaceted modes of action underlying the antagonistic activity of *Bacillus* strains against *A. flavus.*

Strain BAF143 was selected for this study based on the following considerations: (i) it is a marine-derived *Bacillus* strain, isolated from a unique ecological niche, which may produce structurally novel antifungal metabolites; (ii) its crude extract has exhibited potent antifungal activity against *A. flavus*, with high inhibition rates of mycelial growth (62.36 ± 1.00%) and spore germination (90.10 ± 0.21%); (iii) preliminary transcriptomic analysis has revealed a unique multi-pathway compensatory collapse mechanism that differs from previously reported modes of action; and (iv) the crude extract has demonstrated strong biocontrol efficacy on peanuts, achieving a 96.18% reduction in fungal contamination during storage. These features make BAF143 a promising candidate for both mechanistic studies and practical applications in postharvest preservation.

The present study was designed to systematically dissect the antifungal mechanism of a selected antagonistic *Bacillus* strain and its crude extract against *A. flavus*. We hypothesized that the crude extract exerts its fungistatic effect through a multi-pathway attack on fungal cells. To test this hypothesis, we employed a multi-pronged approach: (i) evaluating the impact on mycelial morphology and ultrastructure; (ii) assessing the integrity and permeability of the cell membrane; (iii) quantifying oxidative stress markers and DNA damage; and (iv) integrating transcriptomic profiling (RNA sequencing) to capture the genome-wide transcriptional reprogramming in response to the treatment. Furthermore, we investigated its potential to protect peanuts, a major host crop, from *A. flavus* infection under controlled storage conditions. By integrating physiological and transcriptomic evidence, this work aims to construct a holistic model of the inhibitory action of *Bacillus* metabolites against *A. flavus*. The findings will not only elucidate the intricate molecular interplay between the biocontrol agent and the pathogen but also provide a robust theoretical foundation for the rational design and application of microbial biopreservatives in the agricultural and food industries.

## 2. Results

### 2.1. Screening of Antifungal Bacterial Activity Against A. flavus on Different Culture Media

To rigorously evaluate the antagonistic activity of strain BAF143 against *A. flavus* and, more importantly, to exclude the potential confounding effects of culture media composition on the growth of either the bacterial antagonist or the fungal pathogen, three distinct agar media, including potato dextrose agar (PDA, a standard fungal medium), martin medium (MT, a selective fungal medium), and Luria–Bertani agar (LB, a standard bacterial medium), were employed in a plate standoff assay.

**Figure 1:**
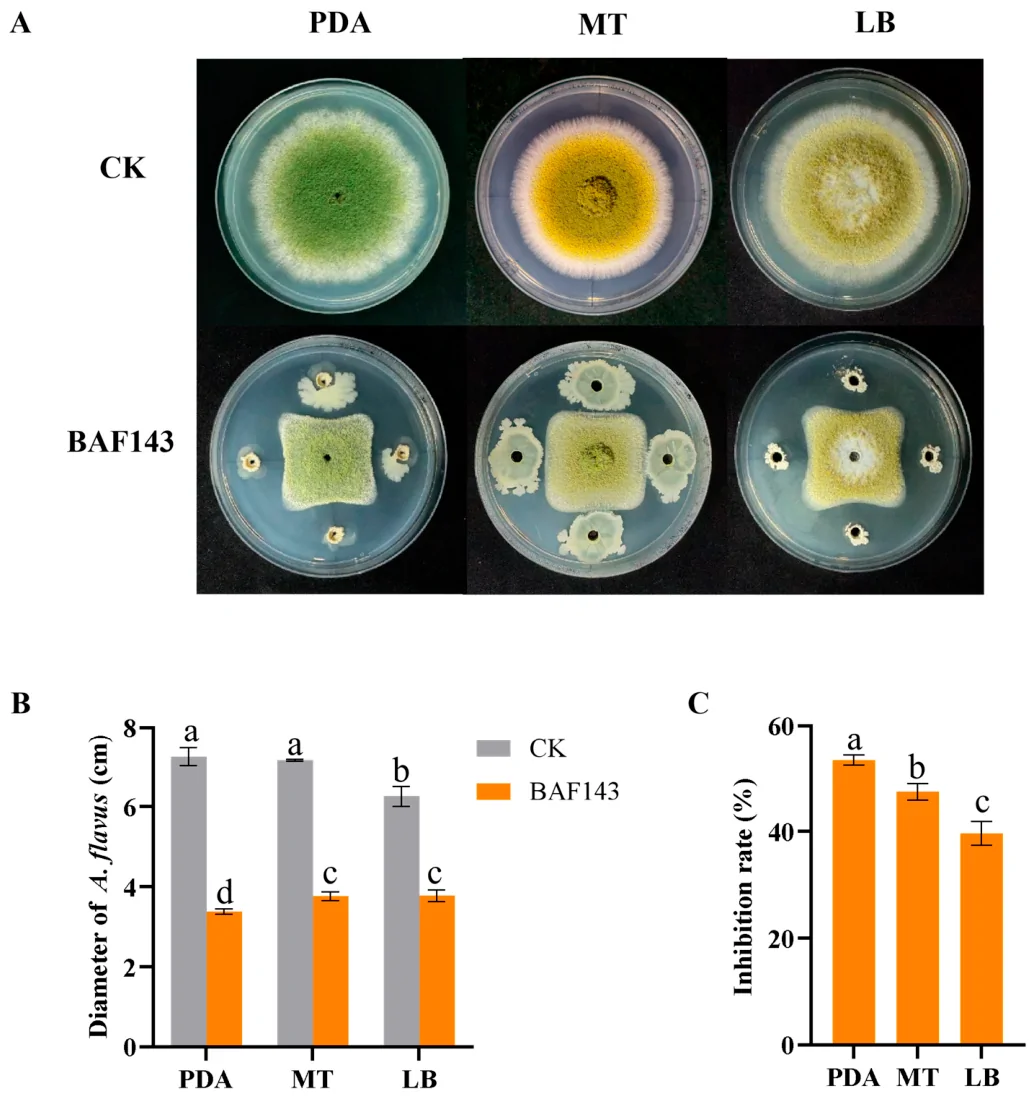
The inhibitory effect of *A. flavus* during plate standoff with *Bacillus* sp. BAF143 in three media. (**A**) Plate confrontation assay between *A. flavus* and *Bacillus* sp. BAF143. (**B**) Colony diameter of *A. flavus* during plate standoff. (**C**) Mycelial inhibition rate of *A. flavus* during plate standoff. Different letters indicate significant differences (*p* < 0.05) according to Tukey’s post hoc test.

As shown in Figure 1, strain BAF143 exhibited observable inhibitory zones against the mycelial growth of *A. flavus* on all three tested media, albeit with markedly different intensities. The strongest antifungal activity was recorded on PDA, whereas the weakest inhibition was observed on LB (Figure 1A). In the control group (without bacterial inoculation), the colony diameters of *A. flavus* reached 7.25 ± 0.23 cm, 7.17 ± 0.03 cm and 6.26 ± 0.23 cm on PDA, MT and LB, respectively (Figure 1B). In the treatment group co-cultured with BAF143, the colony diameters of *A. flavus* were significantly reduced to 3.38 ± 0.07 cm, 3.76 ± 0.11 cm and 3.78 ± 0.14 cm on PDA, MT and LB, respectively (Figure 1B). The corresponding inhibition rates were calculated as 53.42 ± 0.94% (PDA), 47.46 ± 1.54% (MT) and 39.67 ± 2.27% (LB), indicating a clear medium-dependent antagonistic performance (Figure 1C). The differential antifungal activity of strain BAF143 against *A. flavus* across the three tested media (PDA, MT, and LB) indicates that its antagonistic efficacy is strongly influenced by nutritional composition.

### 2.2. Identification of Strain BAF143

**Figure 2:**
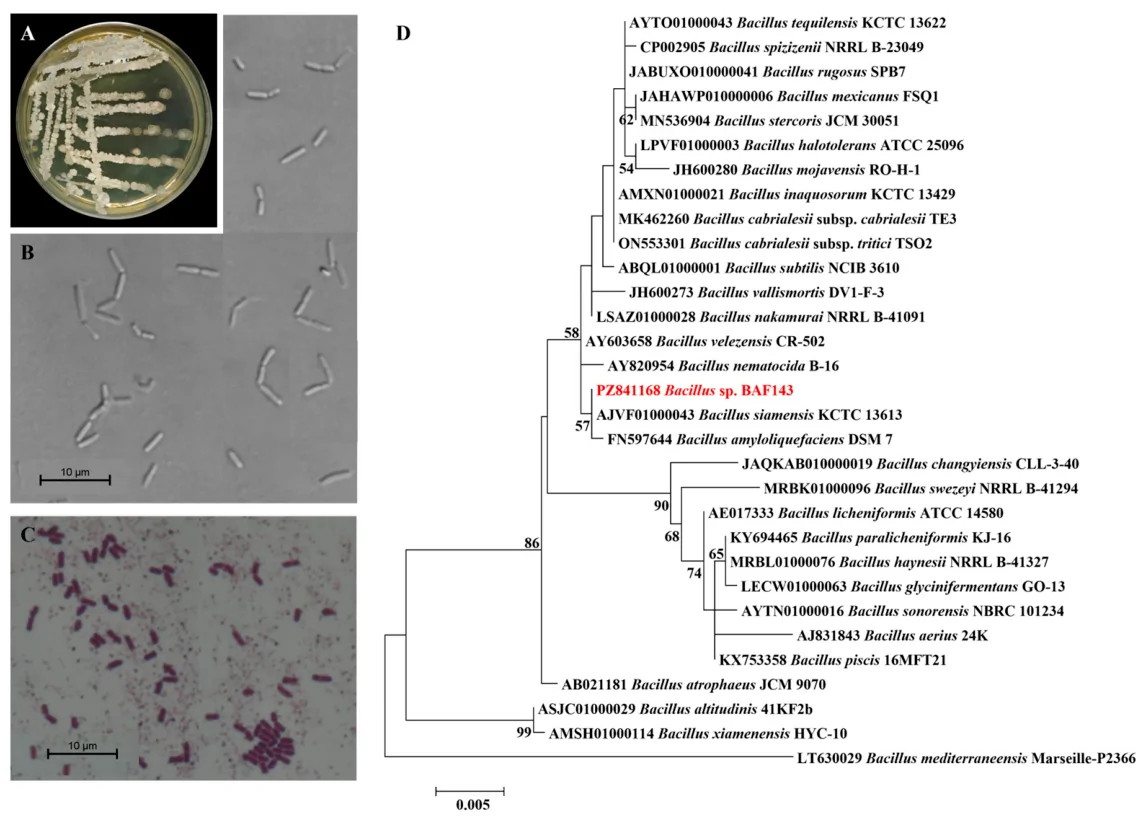
The morphological characteristics and phylogenetic analysis of strain BAF143. (**A**) Colony after 24 h at 30 °C on LB. (**B**) Bacterial morphology image under microscope. (**C**) Gram-staining image under microscope. (**D**) Phylogenetic tree generated based on 16S rRNA gene sequences. The strain marked in red indicates the strain investigated in this study.

The taxonomic position of strain BAF143 was determined through a polyphasic approach combining morphological observation and 16S rRNA gene sequence analysis. The colony morphology of strain BAF143 was milky white on LB medium after 24 h at 28 °C, mucoid, irregularly margined, and large circular (Figure 2A). Under the microscope, the morphology of strain BAF143 was rod shaped (Figure 2B), and Gram staining revealed that it is a Gram-positive bacterium (Figure 2C). The phylogenetic tree (Figure 2D) was established based on the 16S rRNA gene sequences of 31 strains, with LT630029 *Bacillus mediterraneensis* Marseille-P2366 as the outgroup. The 16S rRNA gene sequences of strain BAF143 clustered with *Bacillus siamensis* and *Bacillus amyloliquefaciens* (MLBP = 57%). Based on the integrated phenotypic and genotypic evidence, strain BAF143 was conclusively identified as *Bacillus* sp. BAF143.

### 2.3. Inhibitory Effect of Crude Extract from BAF143 on the Growth of A. flavus

#### 2.3.1. Antifungal Activity of Fermentation Time-Course Extracts Against *A. flavus*

To optimize the fermentation conditions for maximum antifungal metabolite production, strain BAF143 was cultured in liquid LB over a time course, and the crude extracts prepared from fermentation broth harvested at different time points (1, 2, 3, 4, 5, 6, and 7 days) were evaluated for their inhibitory activity against *A. flavus* using the agar well diffusion method.

**Figure 3:**
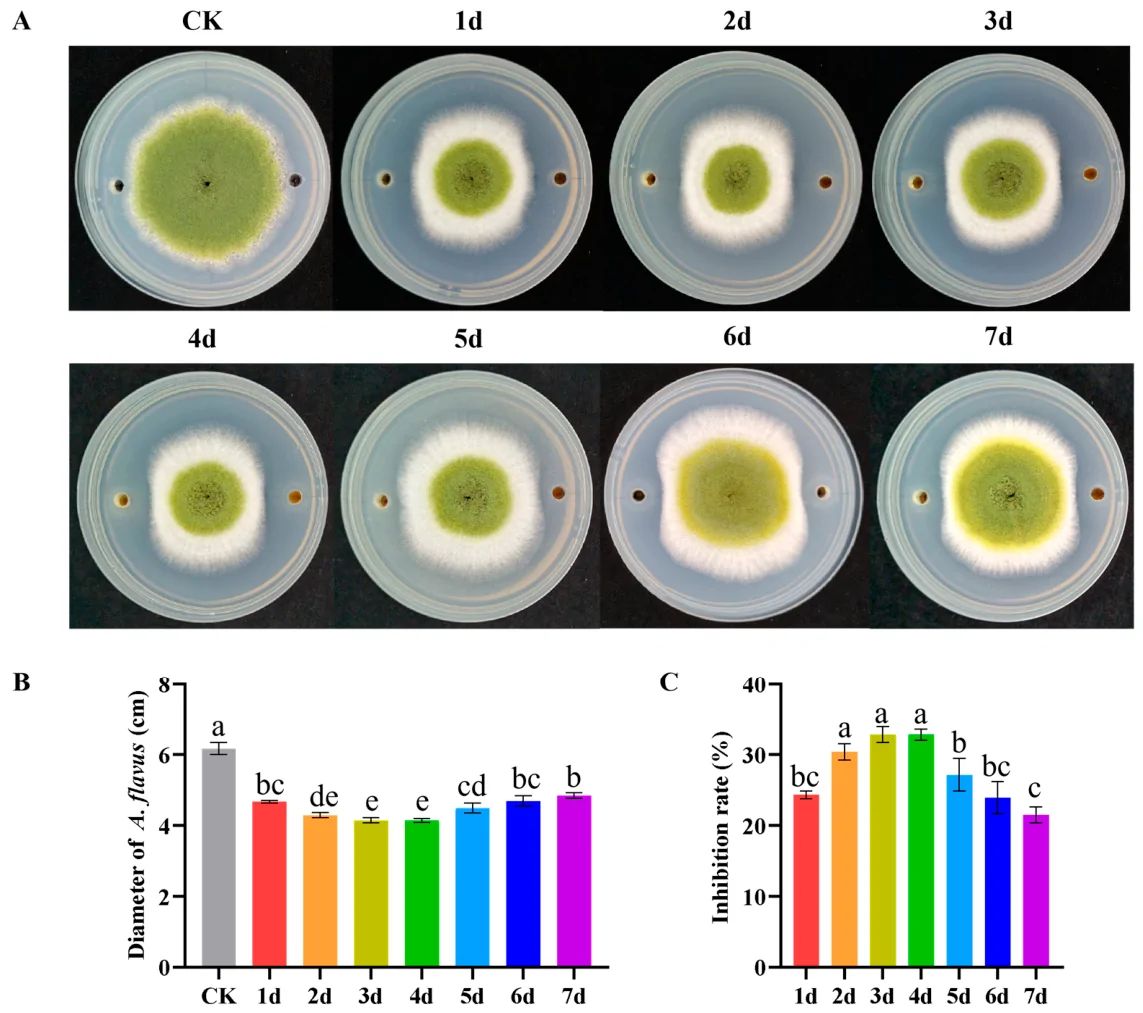
The inhibitory effects of different fermentation times on *A. flavus.* (**A**) The agar well diffusion method. (**B**) Colony diameter of *A. flavus*. (**C**) Inhibition rate of *A. flavus*. Different letters indicate significant differences (*p* < 0.05) according to Tukey’s post hoc test.

As shown in Figure 3A, all seven crude extracts exhibited observable inhibitory effects on the mycelial growth of *A. flavus*. The colony diameter of *A. flavus* reached 6.18 ± 0.17 cm in the control group (with methanol solvent), while the colony diameters of *A. flavus* were reduced to 4.15–4.85 cm in treatment groups (with seven extracts) (Figure 3B). As shown in Figure 3C, the fermentation extracts exhibited time-dependent antifungal activity against *A. flavus*. The inhibition rates increased progressively from 1 day to 4 days of fermentation, reaching a maximum of 32.85 ± 1.15% and 32.85 ± 0.81% at 3 days and 4 days, respectively (Figure 3C). Beyond 4 days, a gradual decline in activity was observed, with the inhibition rate decreasing to 21.52 ± 1.15% at 7 days. Statistical analysis revealed that the 3 days and 4 days extracts exhibited significantly higher activity compared to extracts from other time points (*p* < 0.05), indicating that the optimal fermentation periods for bioactive metabolite production are 3 days and 4 days under the tested conditions. Therefore, in the subsequent experiments, a fermentation time of 3 days was chosen.

#### 2.3.2. Poison Food Technique and Minimum Inhibitory Concentration (MIC) Assay

**Figure 4:**
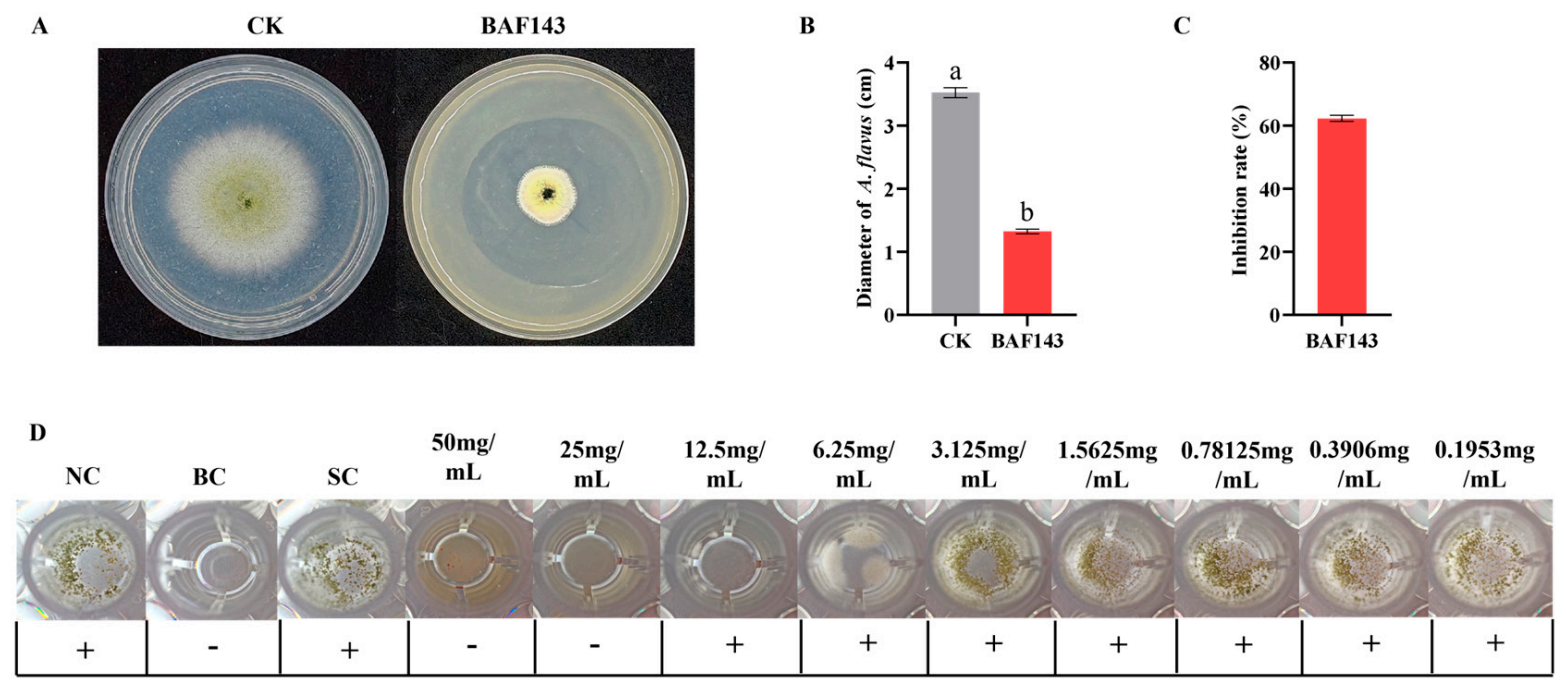
Evaluation of the crude extract from strain BAF143 against *A. flavus* using the poison food technique and minimum inhibitory concentration assay. (**A**) Poison food technique. (**B**) Colony diameter of *A. flavus*. (**C**) Inhibition rate of *A. flavus*. (**D**) MIC determination via 96-well plate broth microdilution assay, NC represents negative control, BC represents blank control, SC represents solvent control. Different letters indicate significant differences (*p* < 0.05) according to Tukey’s post hoc test.

To evaluate the antifungal activity of the crude extract of the BAF143 strain by different methods, the activity was tested using the poison food technique. The crude extract obtained from the optimal 3 days fermentation was incorporated into PDA at 1 mg/mL, with an equivalent volume of the methanol solvent added to the control medium, and the mycelial growth of *A. flavus* was monitored at 5 days for 30°C. In the control group, the colony diameter reached 3.52 ± 0.08 cm (Figure 4A,B). In the treatment group, the colony diameter was reduced to 1.33 ± 0.04 cm (Figure 4A,B), corresponding to an inhibition rate of 62.36 ± 1.00% (Figure 4C).

The MIC was determined using a 96-well microtiter plate assay (Figure 4D). The assay included negative control (broth and spore suspension), blank control (broth only), solvent control (methanol in broth and spore suspension), and treatment (different concentrations of crude extracts in broth and spore suspension). As shown in Figure 4D, the crude extract exhibited a dose-dependent inhibitory effect on the mycelial growth of *A. flavus*. With increasing concentrations of the extract, the mycelial number of *A. flavus* progressively decreased. The concentrations of crude extracts ranged from 0.1953 to 50 mg/mL, and significant mycelial growth inhibition was detected at 6.25 mg/mL, whereas complete suppression of fungal growth was recorded at the maximum concentration of 25 mg/mL.

### 2.4. Inhibitory Effect of Crude Extract from BAF143 on Spore Germination of A. flavus

**Figure 5:**
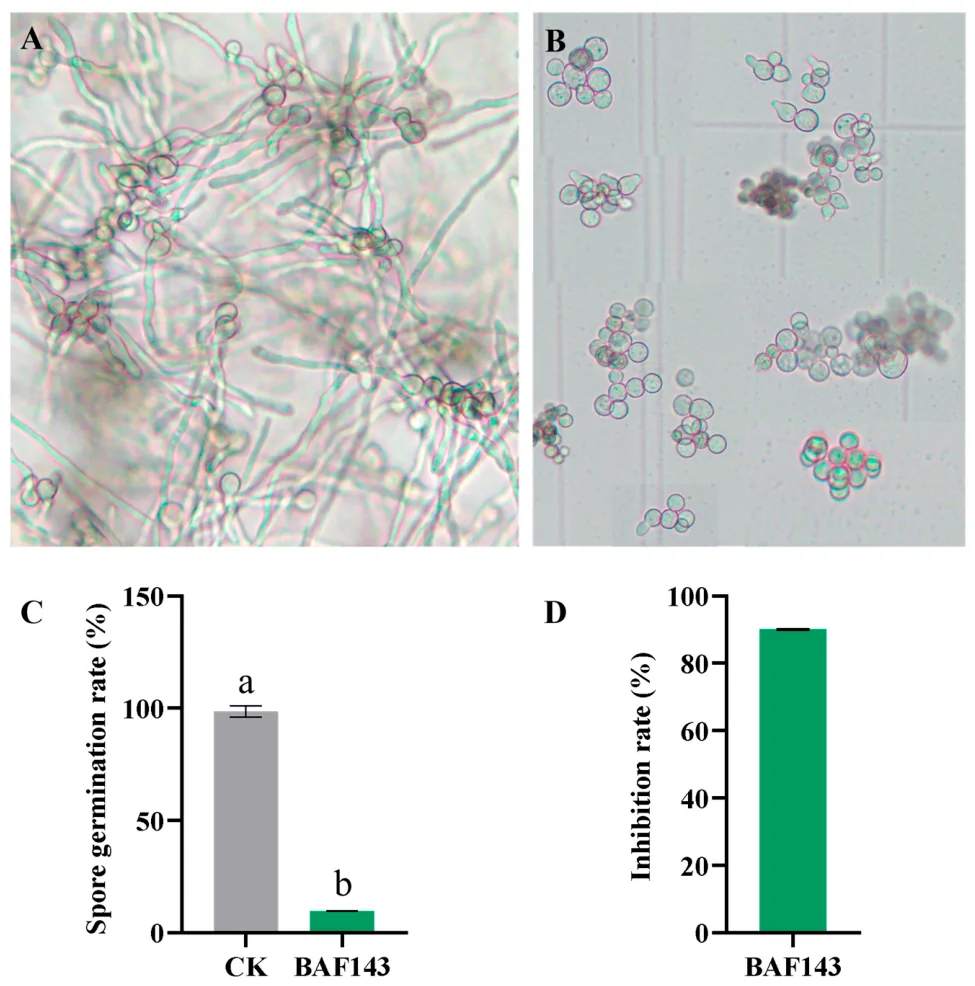
The influence of crude extract of BAF143 on spore germination of *A. flavus*. (**A**) Control (methanol only). (**B**) Spores of *A. flavus* treated with crude extract at 25 mg/mL. (**C**) Spore germination rate of *A. flavus*. (**D**) Inhibition rate of spore germination of *A. flavus*. Different letters indicate significant differences (*p* < 0.05) according to Tukey’s post hoc test.

To further evaluate the antifungal spectrum of the crude extract from strain BAF143, its inhibitory effect on conidial germination of *A. flavus* was assessed. The results of the BAF143 crude extract on conidial germination of *A. flavus* are presented in Figure 5. In the control group, the spores almost completely germinated, with long hyphae clearly visible (Figure 5A), and germination rate reached 98.55 ± 2.52% after 24 h of incubation (Figure 5C), indicating that the spores were highly viable and the solvent had no adverse effect on germination. In the treatment group, spore germination was almost completely suppressed, only a few small protrusions could be seen on the spores (Figure 5B), and germination rate dropped significantly to 9.76 ± 0.21% (Figure 5C), corresponding to an inhibition rate of 90.10 ± 0.21% (Figure 5D). The solvent control showed no observable effect on spore germination, confirming that the inhibitory activity was solely attributable to the bioactive compounds present in the crude extract.

### 2.5. Effect of Crude Extract on A. flavus Mycelial Morphology and Ultrastructure

**Figure 6:**
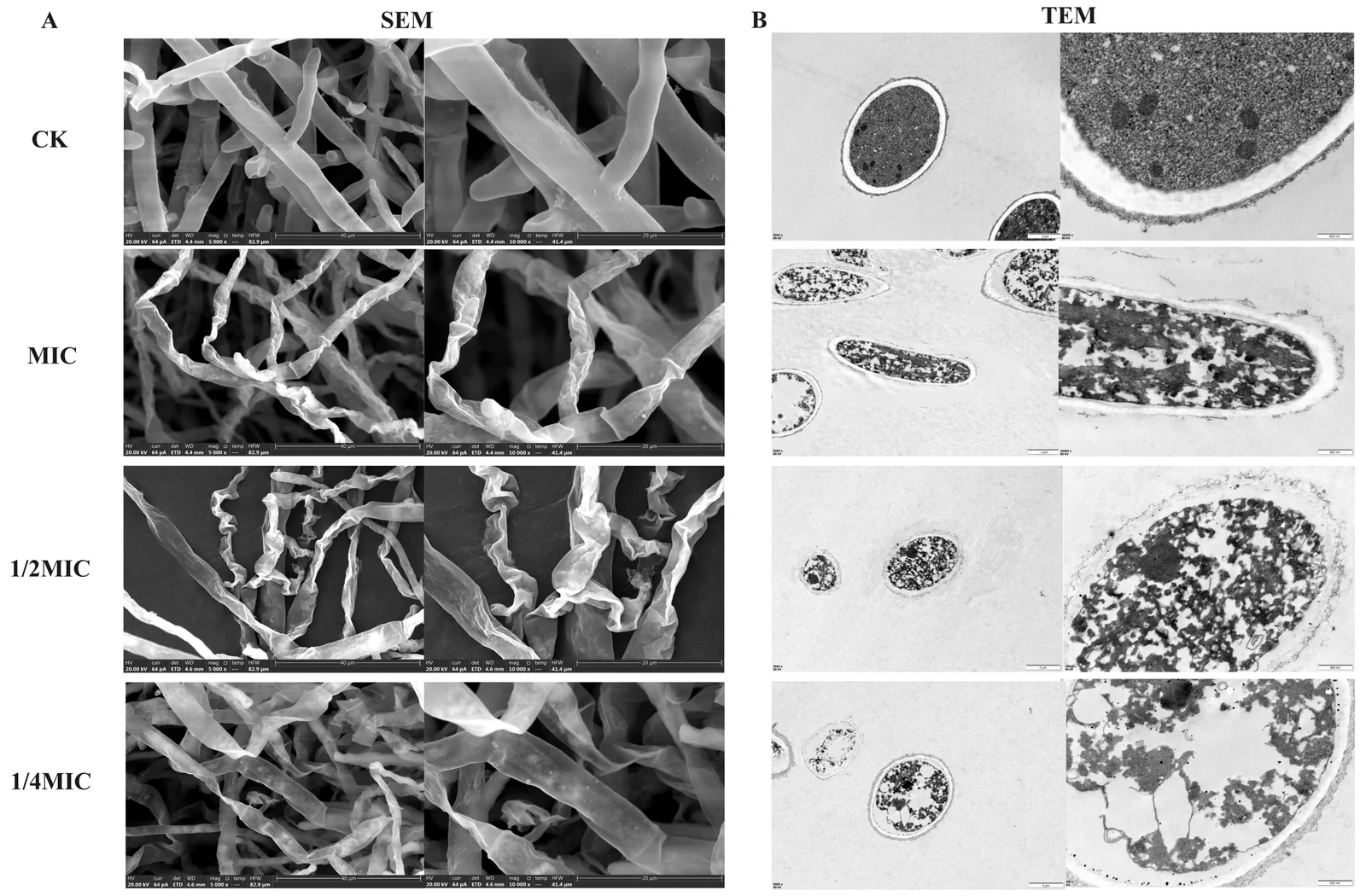
SEM (**A**) and TEM (**B**) images of mycelial morphology of *A. flavus* treated with crude extract of *Bacillus* sp. BAF143 at 0, MIC (25 mg/mL), 1/2 MIC (12.5 mg/mL), and 1/4 MIC (6.25 mg/mL).

To ascertain whether the bacterial crude extract exerts effects on mycelial morphology and ultrastructure in addition to its inhibitory effect on mycelial growth, we conducted SEM and TEM examinations. As show in Figure 6A, the mycelia in the control group displayed typical features of healthy fungal hyphae, including straight, regular, round and smooth surfaces with intact and turgid morphology, without any visible folds, ruptures, or deformations. Conidiophores appeared normally developed. In contrast, the mycelia treated with the crude extract at 25 mg/mL (MIC), 12.5 mg/mL (1/2 MIC) and 6.25 mg/mL (1/4 MIC) exhibited severe morphological abnormalities (Figure 6A). The hyphae appeared shrunken, distorted, twisted and collapsed, with rough and wrinkled surfaces. Notably, some hyphae showed complete collapse, indicating a loss of structural integrity. In addition to hyphal damage, markedly fewer conidiophores were observed on the treated hyphae compared to the control (Figure 6A). A clear dose–response relationship was observed, with the severity of morphological damage progressively increasing from 6.25 to 25 mg/mL. The progressive morphological deterioration from mild surface roughening at 1/4 MIC to complete structural collapse at MIC demonstrates the concentration-dependent nature of the antifungal activity.

TEM further revealed the ultrastructural damage caused by the crude extract. In the control group (Figure 6B), *A. flavus* mycelia displayed a well-defined cellular ultrastructure with a smooth and continuous cell wall, an intact plasma membrane, and a homogeneous cytoplasm containing well-preserved organelles. The cell wall appeared uniformly electron dense with a distinct and continuous outer layer. However, in the treatment group (Figure 6B), the cell wall became irregular, loose and thinned or ruptured, with the outer layer almost entirely degraded or detached. The plasma membrane was detached from the cell wall, with invaginations and discontinuities observed in many regions, indicating compromised membrane integrity. Extensive leakage of cytoplasmic contents caused a marked decrease in intracellular material, accompanied by profound disorganization of the cytoplasmic architecture and the loss of identifiable organelles.

### 2.6. Transcriptomic Analysis

**Figure 7:**
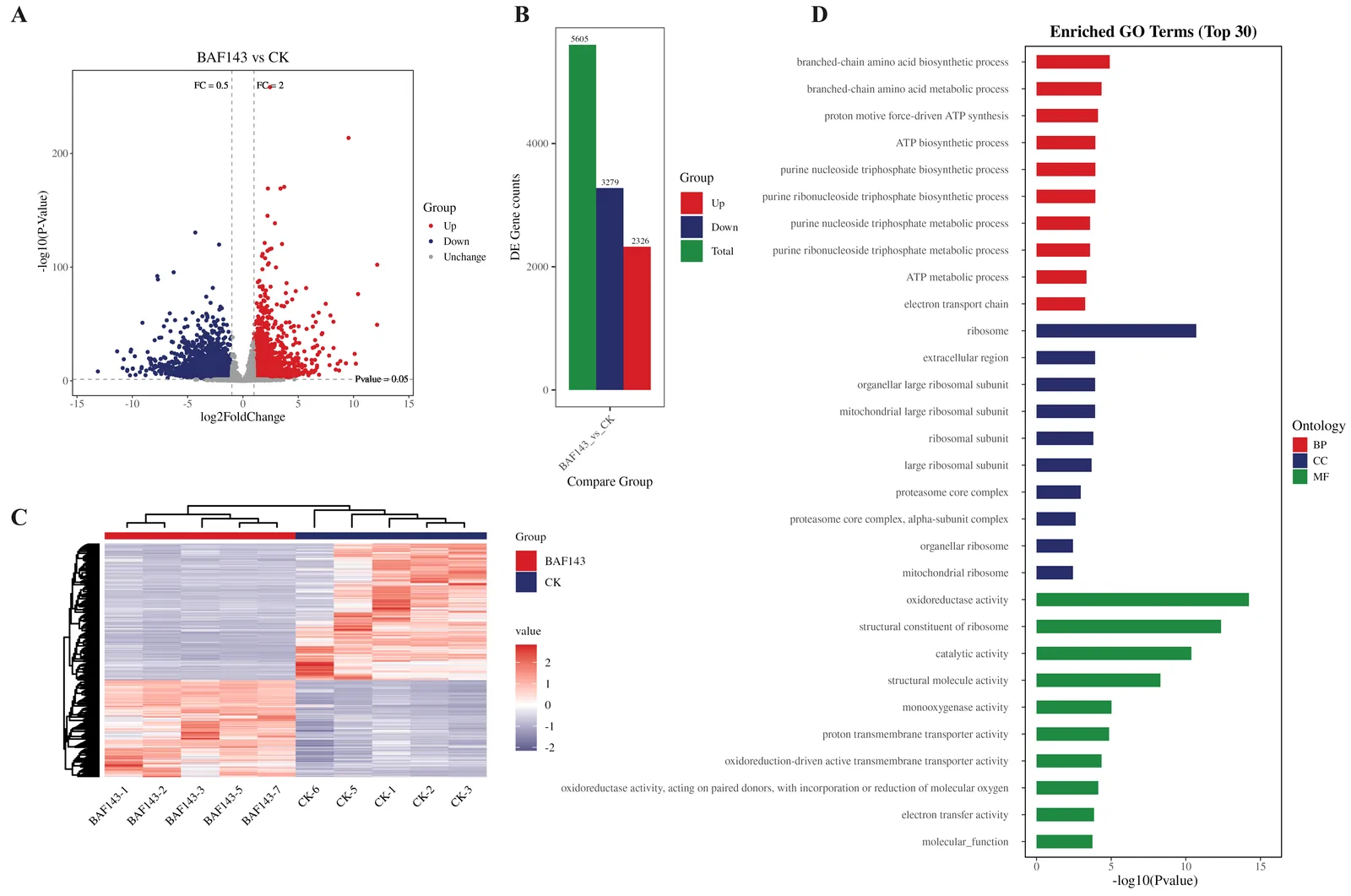
Transcriptomic analysis of *A. flavus* mycelium under CK and treatments. (**A**) Volcano plot of DEGs. (**B**) Statistical bar of DEGs. (**C**) Heatmap of DEGs. (**D**) GO enrichment analysis of DEGs.

To elucidate the molecular mechanism underlying the antifungal activity of the BAF143 crude extract against *A. flavus*, transcriptomic analysis was performed to capture the genome-wide transcriptional reprogramming in response to the treatment. Transcriptomic analysis revealed a total of 5605 differentially expressed genes (DEGs) between the crude extract-treated and control groups (Figure 7A). Among these, 2326 genes were significantly upregulated, while 3379 genes were significantly downregulated in response to the treatment (Figure 7B). A heatmap (Figure 7C) displayed distinct expression patterns between the two groups, indicating that the crude extract induced profound transcriptional reprogramming in *A. flavus*. GO enrichment analysis was performed to classify the DEGs into three functional categories: biological process (BP), cellular component (CC), and molecular function (MF) (Figure 7D). The most enriched functions included oxidoreductase activity, structural constituent of ribosome, ribosome, catalytic activity, and structural molecule activity. This suggests that *A. flavus* mounted a robust stress response to the oxidative damage, impaired protein biosynthesis, and compromised membrane integrity induced by the crude extract.

**Figure 8:**
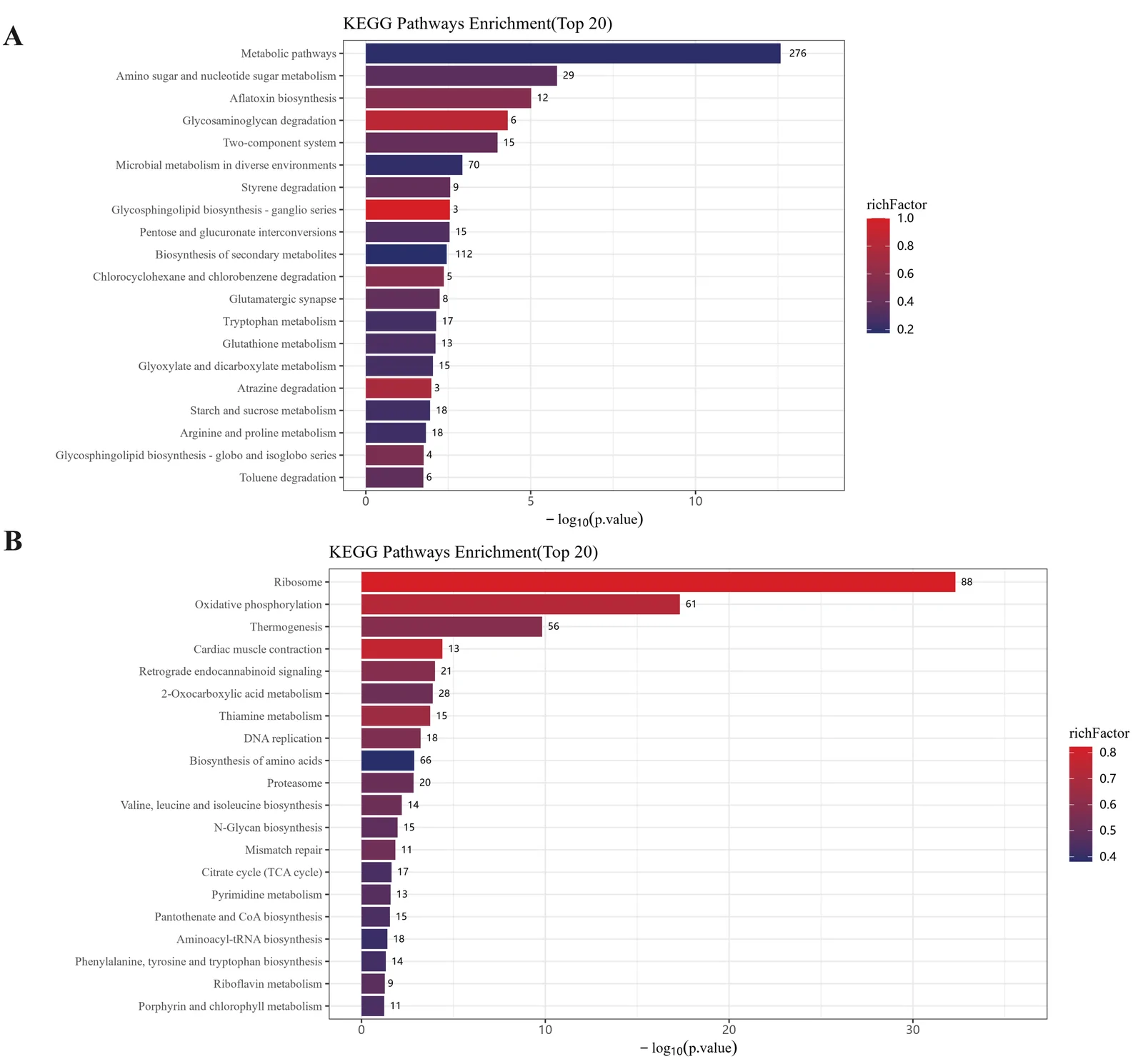
KEGG enrichment analysis of DEGs. (**A**) The downregulated DEGs. (**B**) The upregulated DEGs.

KEGG pathway enrichment analysis revealed that downregulated genes were predominantly enriched in metabolic pathways (276 genes), indicating a global suppression of anabolic processes (Figure 8A). Notably, the downregulation of amino sugar and nucleotide sugar metabolism, which is involved in cell wall component synthesis (chitin and glucans), was consistent with the hyphal morphological damage observed by SEM/TEM. Among upregulated genes, the most enriched pathways were ribosome (88 genes), oxidative phosphorylation (61 genes), and thermogenesis (56 genes) (Figure 8B). The upregulation of ribosome and proteasome pathways suggests a compensatory response to protein damage, while the enrichment of oxidative phosphorylation and thermogenesis points to an attempt to sustain ATP production under stress. Additionally, the upregulation of DNA replication and mismatch repair pathways implies that the crude extract may induce genotoxic stress.

To further characterize the impact of the crude extract on *A. flavus* mycelial growth, representative DEGs were classified into eight functional categories, including cell wall synthesis, ergosterol synthesis, antioxidant enzymes, cell cycle, DNA replication, ribosomal proteins, energy metabolism and MAPK signaling pathways (Table 1). Downregulation of chitin synthase genes compromised cell wall integrity, while the upregulation of ergosterol biosynthesis genes likely reflected a compensatory response to reinforce membrane integrity. The bidirectional regulation of antioxidant genes induced oxidative stress, with early activation followed by eventual exhaustion of antioxidant capacity. Upregulation of cell cycle and DNA replication genes indicated a paradoxical attempt to drive mitotic progression and S-phase entry, which, under severe cell wall and membrane damage, may lead to mitotic catastrophe and accelerate cell death. Concurrently, the upregulation of ribosomal protein and energy metabolism genes represented a futile attempt to sustain protein synthesis and ATP production, given the mitochondrial damage observed by TEM. Notably, the downregulation of MAPK signaling pathway genes impaired fungal stress sensing and compromised cell wall repair capacity. Collectively, these transcriptional changes depict a multifaceted antifungal mechanism, wherein the crude extract simultaneously disrupts cell wall integrity, induces oxidative stress, impairs energy homeostasis, and dampens stress signaling, ultimately leading to the collapse of cellular homeostasis and growth inhibition of *A. flavus*.

### 2.7. Effect of Crude Extract on the Integrity of Cell Membrane and MMP of A. flavus Mycelia

**Figure 9:**
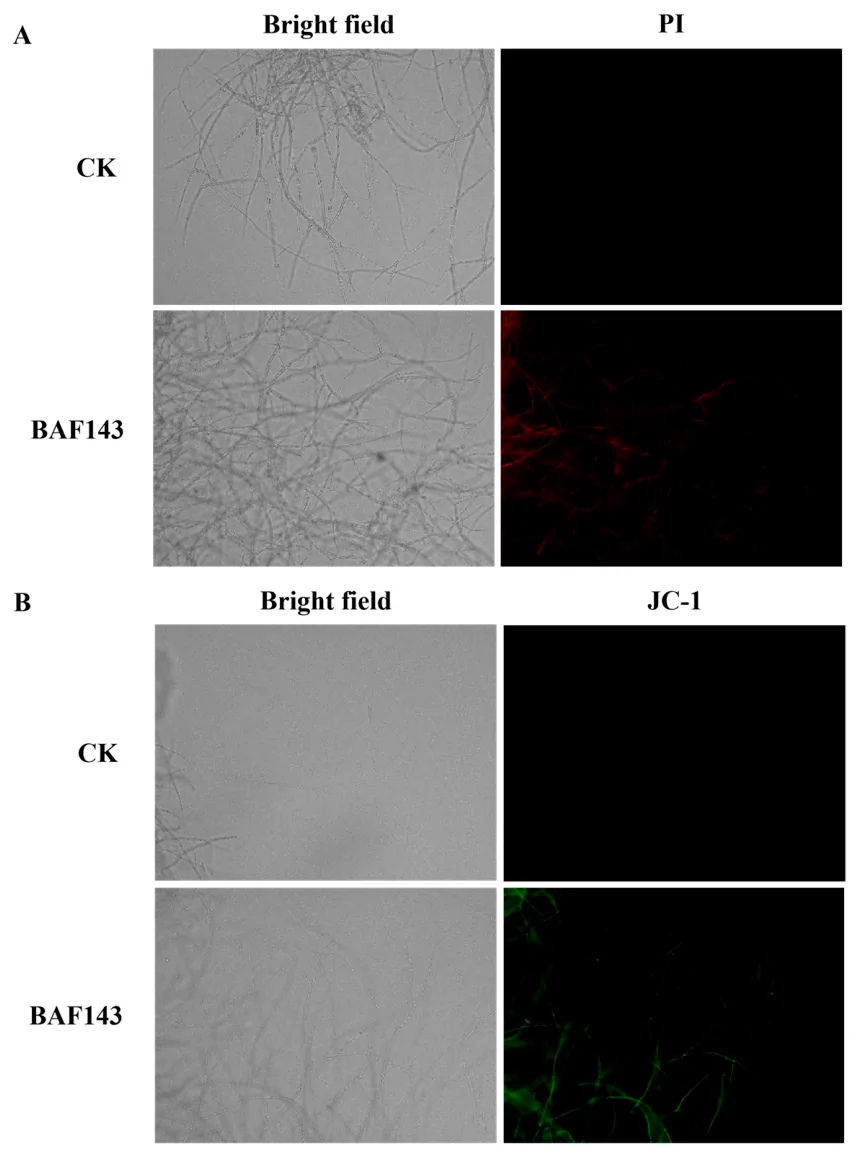
Crude extract of BAF143 induces cell membrane integrity and mitochondrial function damage in *A. flavus*. (**A**) Fluorescence microscopic analysis of *A. flavus* cell membrane after PI staining. (**B**) Fluorescence microscopic analysis of *A. flavus* mitochondria after JC-1 staining.

Based on the transcriptomic data, which showed downregulation of cell wall synthesis-related genes and concomitant upregulation of key genes in the ergosterol biosynthetic pathway, we inferred that *A. flavus* might mount a compensatory response to counteract cell wall damage. To test this hypothesis, we subsequently examined the effects of the crude extract on cell membrane integrity and MMP. The effect of the crude extract on *A. flavus* cell membrane integrity was evaluated by PI staining (Figure 9A). In the control group, no mycelia exhibited red fluorescence, indicating that the majority of cells maintained intact membranes. However, treatment with 25 mg/mL crude extract resulted in strong red fluorescence, demonstrating extensive and severe membrane damage affecting the vast majority of cells.

The effect of the crude extract on MMP was assessed using the JC-1 probe (Figure 9B). No green fluorescence was observed in the control group, indicating high MMP (normal mitochondrial function). Whereas after treatment with 25 mg/mL crude extract, the mycelia exhibited strong green fluorescence, indicating severe mitochondrial membrane depolarization and loss of mitochondrial function. These results indicate that the crude extract of BAF143 significantly damaged cell membrane integrity and mitochondrial dysfunction.

### 2.8. Effect of Crude Extract on Antioxidant and DNA Damage of A. flavus Mycelia

**Figure 10:**
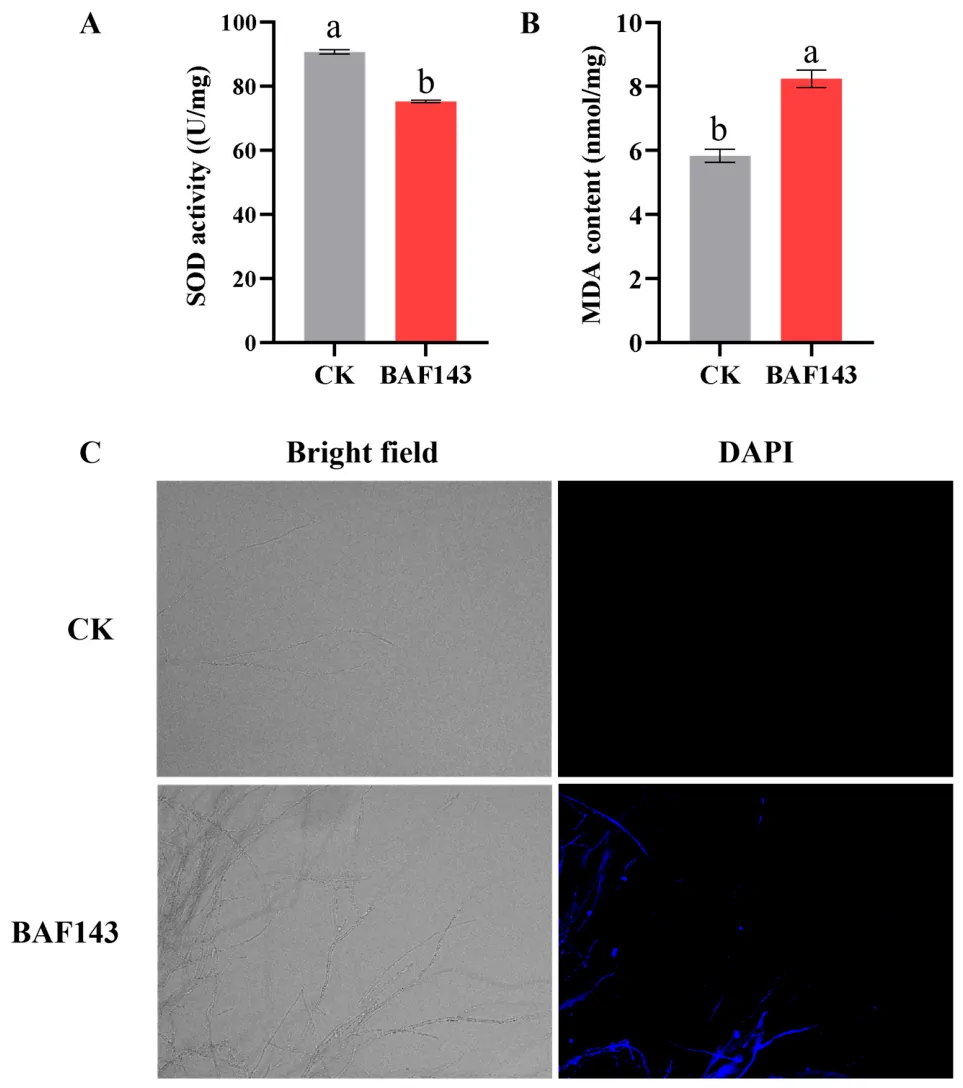
Effect of crude extract of BAF143 on the antioxidant system and DNA damage. (**A**) SOD activity of *A. flavus*. (**B**) MDA content of *A. flavus*. (**C**) DAPI staining. Different letters indicate significant differences (*p* < 0.05) according to Tukey’s post hoc test.

The activities of SOD showed distinct response patterns (Figure 10A). The SOD activity in *A. flavus* mycelia reached 90.74 ± 0.72 U/mg protein in the control group. However, upon treatment with the crude extract at 25 mg/mL (MIC), SOD activity reduced to 75.32 ± 0.42 U/mg protein, representing a reduction of 16.99% compared to the control. The results indicated that crude extract of BAF143 caused the antioxidant capacity of *A. flavus* to diminish. The MDA content, a marker of lipid peroxidation, increased following treated with crude extract of BAF143 (Figure 10B). In the control group, the MDA content was 5.83 ± 0.21 nmol/mg protein. At MIC (25 mg/mL), MDA levels increased to 8.25 ± 0.27 nmol/mg protein, representing a 1.4-fold increase over the control. The elevated MDA levels indicate extensive lipid peroxidation of cell membranes, which likely contributed to the membrane disruption.

Nuclei were stained with DAPI for visualization of nuclear morphology, which help to assess cell damage and apoptotic-like changes. No blue fluorescence signal appeared in *A. flavus* mycelia in the control group, indicating that the nuclei remained intact (Figure 10C). In contrast, intense blue fluorescence was observed in the treatment group (Figure 10C), suggesting that the crude extract of BAF143 destroyed the membrane permeability of *A. flavus*, caused further nuclear damage and chromatin condensation, and induced apoptosis-like cell injury.

### 2.9. Crude Extract Inhibited the Infection of A. flavus on Peanuts

**Figure 11:**
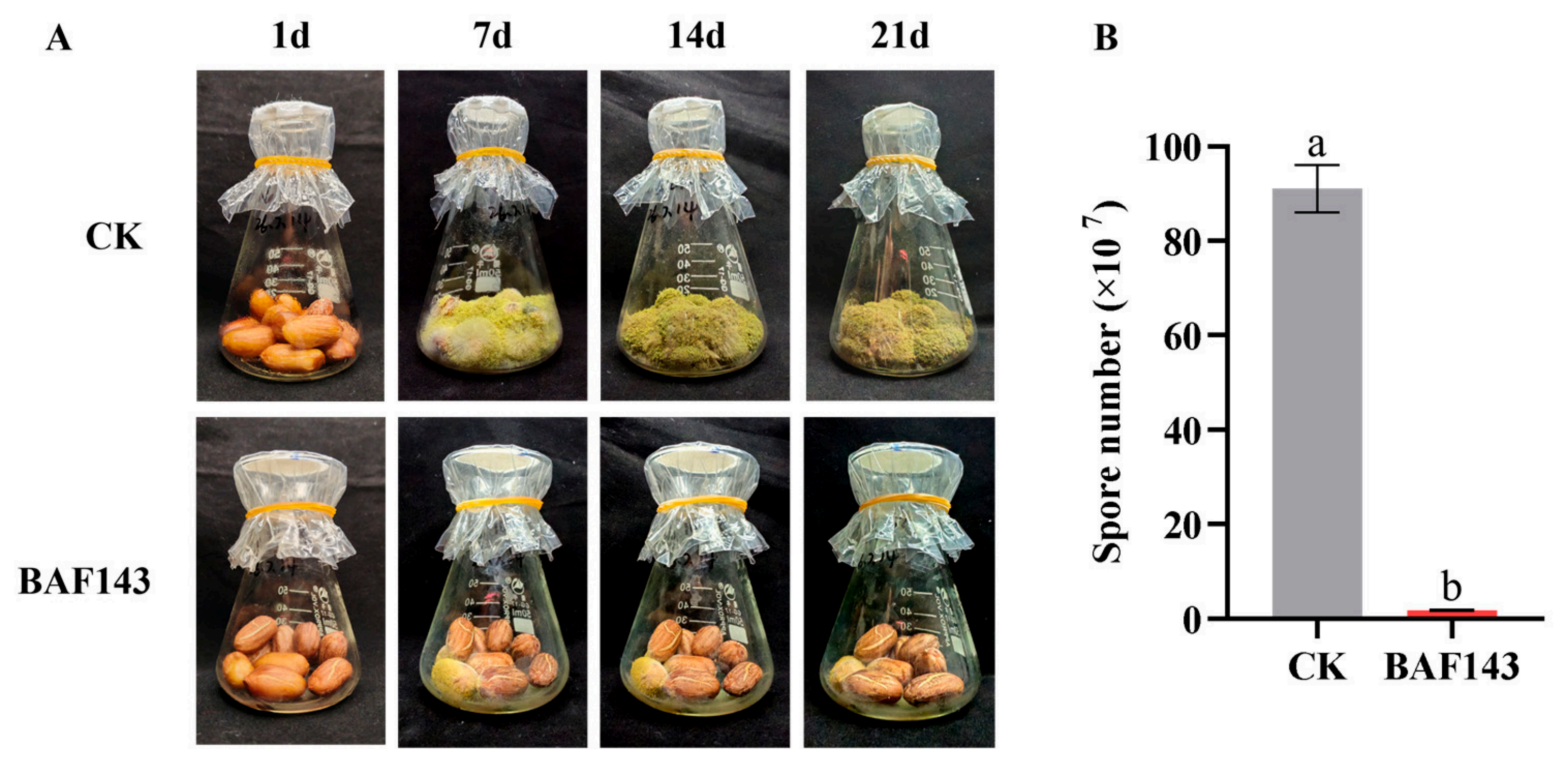
Effect of crude extract from BAF143 on *A. flavus* infection on peanuts. (**A**) Morphology of peanuts with *A. flavus* after 1 day, 7 days, 14 days, 21 days of incubation at 30 °C with control (methanol only) and 25 mg/mL (MIC) crude extract. (**B**) The number of spores of *A. flavus* from infected peanuts after 21 days. Different letters indicate significant differences (*p* < 0.05) according to Tukey’s post hoc test.

To investigate the protective effect of the BAF143 crude extract against *A. flavus* infection, peanut kernels were treated with the extract at 25 mg/mL and incubated at 30 °C for up to 21 days, with untreated kernels serving as the control (Figure 11). Time-course analysis revealed that the control group displayed a continuous and progressive increase in fungal contamination over the incubation period (Figure 11A). In marked contrast, the treatment group exhibited only a transient increase in contamination during the initial 7 days, after which the fungal load remained essentially unchanged. Quantitative analysis at 21 days post incubation showed that the spore population in the control group reached (91.00 ± 5.00) × 10^7^, whereas that in the treatment group was only (1.77 ± 0.13) × 10^7^, indicating a substantial reduction of 96.18% (Figure 11B). These findings demonstrate that the crude extract from *Bacillus* sp. BAF143 has strong potential as a biocontrol agent for preventing *A. flavus* contamination in post-harvest agricultural products.

## 3. Discussion

The antagonistic activity of strain BAF143 against *A. flavus* was strongly influenced by nutritional composition, with the highest inhibition rate (53.42%) observed on PDA, suggesting that the biosynthesis of antifungal metabolites is nutrition inducible. This phenomenon is consistent with previous reports on *Bacillus* and *Pseudomonas* species, where the expression of secondary metabolite biosynthetic gene clusters is tightly regulated by carbon-to-nitrogen ratios and specific carbohydrate sources [36,37]. The significantly lower activity on LB (39.67%) implies that high peptone and salt content may repress the expression of antifungal biosynthetic pathways, as many microbial secondary metabolites are not constitutively produced, but rather induced under stress or nutrient-limited conditions [38]. The intermediate effect on MT (47.46%) further supported a medium-dependent metabolic shift in the production of bioactive compounds. Importantly, the reduced inhibition rate on LB cannot be solely attributed to the suboptimal growth of *A. flavus* on this medium, confirming that BAF143’s antagonistic effect is actively mediated by its metabolic output rather than passive nutrient competition. From an ecological and biocontrol perspective, the enhanced activity on PDA, a medium mimicking fungal-rich environments, suggests that BAF143 is particularly well-equipped to compete with *A. flavus* in natural habitats such as plant surfaces or stored grains. Collectively, these findings establish PDA as the optimal medium for subsequent bioassay-guided fractionation and fermentation optimization.

It is worth noting that the antifungal activity of BAF143 against *A. flavus* varied depending on the assay method employed. The plate confrontation assay (co-culture) yielded a 53.42 ± 0.94% inhibition rate, while the agar well diffusion assay and the poison food technique with the crude extract yielded 32.85 ± 0.81% and 62.36 ± 1.00% inhibition rates, respectively. The lower inhibition rate in the agar well diffusion assay was likely due to the limited diffusion of the crude extract through the agar medium. In contrast, the poison food technique, which ensures uniform contact between the extract and the fungal mycelium, revealed a 62.36 ± 1.00% inhibition rate, comparable to or even higher than that of the co-culture assay. These results indicate that the crude extract of BAF143 possesses potent intrinsic antifungal activity that is independent of microbial competition, and that the antifungal metabolites produced under standard monoculture conditions are sufficient for effective biocontrol applications.

The 16S rRNA gene sequence similarity (>97%) to the type strain strongly supports its species-level assignment, as this threshold is widely accepted for bacterial species delineation [39]. The phenotypic traits observed in this study are also consistent with those previously reported for members of this genus, particularly regarding milky white, mucoid, irregularly margined, and large circular colonies [40]. Considering the clear phylogenetic placement and distinct phenotypic profile, strain BAF143 represents a promising candidate for further investigation into its antifungal mechanisms. Time-course fermentation profiling of strain BAF143 revealed that maximal antifungal activity was achieved at 3 days of cultivation, suggesting that the biosynthesis of antifungal compounds is growth-associated, likely reaching peak production during the late exponential or early stationary phase of bacterial growth [41]. The subsequent decline in activity beyond 4 days may be attributed to several factors, including nutrient depletion in the fermentation medium, autolytic degradation of bioactive compounds by extracellular enzymes, or pH-mediated instability of the metabolites [36,42]. Alternatively, the downregulation of biosynthetic gene clusters encoding antifungal lipopeptides or polyketides could occur as the culture enters the death phase [43,44]. These results establish 3 days as the optimal fermentation duration for obtaining the most potent crude extract, and the time-course data further suggest that the active compounds are likely secondary metabolites, consistent with the fermentation kinetics of well-known biocontrol agents [37].

The crude extract exhibited potent antifungal activity with an MIC of 25 mg/mL, which is notably lower than the 0.512 g/mL reported for other *Bacillus* crude extracts [28] and the 32 mg/well reported for synthetic peptides from *B. megaterium* [18]. This relatively low MIC suggests that BAF143 produces a substantial quantity of highly active antifungal compounds, making it a promising candidate for biocontrol applications. Furthermore, the extract achieved a spore germination inhibition rate of 90.10 ± 0.21%, which is notably higher than the 80% inhibition rate reported for *B. velezensis* cell-free supernatant [30] and exceeds the 60% inhibition rate reported for *B. subtilis* B-FS06 with 25% cell-free culture filtrate [29]. This value is comparable to the 96.63% inhibition achieved with purified bacillomycin D from *B. subtilis* fmbJ at 200 μg/mL [45]. Notably, our result was obtained using a crude extract rather than purified compounds, highlighting the high potency of the BAF143 metabolites and suggesting that multiple bioactive components may act synergistically to achieve this level of inhibition.

The SEM and TEM observations revealed severe morphological and ultrastructural damage in *A. flavus* mycelia treated with the crude extract, including hyphal shrinkage and collapse, surface wrinkling, cell wall thinning and rupture, plasma membrane detachment, extensive cytoplasmic vacuolation, and mitochondrial swelling with cristae loss. These abnormalities are indicative of a profound and irreversible disruption of cellular integrity. These findings are consistent with previous reports on other antifungal metabolite-producing bacteria. Bacillomycin D from *Bacillus subtilis fmbJ* caused damaged, irregular, deformed and distorted hyphae, with the dissolution, thinning, and lumpy appearance of the cell wall, along with the irregularity of the plasma membrane and degeneration of organelles, culminating in the formation of empty holes within the hyphae [45]. Similarly, Li et al. (2026) demonstrated that *Bacillus velezensis*-derived methylmalonic acid induced concentration-dependent damage in *A. flavus*, characterized by hyphal flattening, cell wall wrinkling and thinning, loosening of the fibrous layer, and progressive mitochondrial swelling [6]. SEM and TEM observations also demonstrated that iturin A from *Bacillus* induced severe morphological damage to *A. niger* mycelia, characterized by twisting, irregular folding, surface drying, and depression, which were presumably attributable to cytomembrane disruption and subsequent leakage of intracellular inclusions [46]. The consistency of these observations across multiple studies supports the notion that cell wall and membrane disruption is a conserved antifungal mechanism among *Bacillus*-derived metabolites.

Transcriptomic analysis revealed that the BAF143 crude extract exerts a multifaceted antifungal effect against *A. flavus*, which is generally consistent with previous reports on *Bacillus*-derived metabolites but also exhibits several distinctive features. As in earlier studies [47], we observed significant downregulation of genes involved in cell wall synthesis, including chitin synthases and β-glucan synthases, supporting the notion that suppression of cell wall integrity is a conserved mechanism among antagonistic *Bacillus* strains. Similarly, *Bacillus amyloliquefaciens* SC-B15 was reported to affect the synthesis of chitin and cell wall glucan by controlling genes such as *FksA*, *ChsA*, and *Cat1* [48]. However, our data also revealed unique transcriptional responses. While most studies report downregulation of ribosomal genes under stress conditions, we observed a marked upregulation of ribosomal protein genes (88 genes), which, together with the upregulation of proteasome and energy metabolism pathways, suggests a desperate compensatory effort to sustain protein synthesis and ATP production under stress, an interpretation supported by TEM evidence. Furthermore, the downregulation of the MAPK signaling pathway, coupled with the paradoxical upregulation of ergosterol biosynthesis genes, indicates that the crude extract impairs stress sensing while simultaneously triggering a compensatory membrane reinforcement response that bypasses the canonical CWI–MAPK axis. The bidirectional expression of antioxidant genes (*SOD* and *CAT*) further implies a temporal progression from early oxidative stress activation to eventual exhaustion of antioxidant capacity, likely culminating in irreversible oxidative damage. Collectively, these findings depict a “multi-pathway attack” model in which the BAF143 crude extract simultaneously compromises cell wall integrity, induces mitochondrial dysfunction, depletes energy reserves, and impairs stress signaling, ultimately overwhelming the fungal defense system and leading to growth inhibition and cell death.

Physiological assays provided direct evidence supporting the transcriptomic findings. The increase in PI-positive cells and reduction in MMP confirmed membrane disruption and mitochondrial dysfunction. This membrane-disrupting activity is consistent with multiple previous reports on *Bacillus*-derived antifungal metabolites. Iturin A, a well-characterized lipopeptide from *B. amyloliquefaciens* BH072, induced concentration-dependent disruption of cell membrane integrity in *A. niger* [46]. Similarly, the antimicrobial protein from *Bacillus tequilensis* XZ4-1 was shown to disrupt fungal membrane integrity in *A. flavus* [31], and the active antifungal substances from *B. velezensis* ATC-AL were shown to disrupt fungal membrane integrity, induce ROS accumulation and lipid peroxidation, and collapse mitochondrial membrane potential [49]. Furthermore, the cell-free supernatants of *B. albus* strains were reported to suppress *A. flavus* through synergistic membrane disruption and direct antibiosis [26], and the lipopeptides produced by *B. velezensis* E2, identified as fengycin and iturins, also caused abnormal hyphal enlargement and cell rupture [50]. Collectively, these studies, together with our findings, support the notion that disruption of cell membrane integrity is a conserved and potent antifungal mechanism among *Bacillus* spp., often acting in concert with oxidative stress and mitochondrial dysfunction to achieve fungal inhibition.

The significant reduction in SOD activity (16.99% at MIC) and increase in MDA content (1.4-fold) are consistent with previous studies on *Bacillus*-derived antifungal metabolites. Treatment of *A. flavus* with antimicrobial protein from *Bacillus tequilensis* XZ4-1 resulted in decreased SOD activity and increase in MDA content [31]. Similarly, iturin A produced by *Bacillus amyloliquefaciens* and *Bacillus subtilis* WL-2 was shown to induce a significant increase in MDA content at ½ MIC, and 2 MIC for 12 h [47,51]. The significant ROS accumulation observed in our study indicates that the crude extract induces robust oxidative stress in *A. flavus*, likely originating from mitochondrial dysfunction, where damaged mitochondria become inefficient in electron transfer and generate excessive superoxide radicals. This is consistent with the upregulation of oxidative phosphorylation genes in the transcriptomic data, suggesting that the attempted increase in mitochondrial respiration leads to a “leaky” electron transport chain and subsequent ROS production. DAPI staining further revealed chromatin condensation and nuclear fragmentation, indicating apoptosis-like cell death consistent with reports on 2-ethylhexanol, a VOC produced by some microorganisms [52], and antimicrobial protein from *B. tequilensis* XZ4-1 [31]. The correlation between lipid peroxidation and membrane disruption, together with the demonstration of apoptosis-like nuclear damage, adds novel insights to the understanding of Bacillus-mediated antifungal activity.

The biocontrol efficacy of the BAF143 crude extract was validated on peanuts, achieving a 96.18% reduction in *A. flavus* spore count after 21 days at 25 mg/mL. This is comparable to the 97.93% inhibition reported for *B. siamensis* 3BS12-4 extracellular compounds [28] and superior to the 45.9-65.8% disease control efficacy reported for *B. albus* strains NNK24 and NDP6 using cell-free supernatants [26], the 29.2% reduction in grape infection rate by *B. tequilensis* XZ4-1 antimicrobial protein [31], and the 19-47% reduction on maize grains treated with bacterial suspensions from *Bacillus safensis* RF69, *Bacillus* sp. RP103, and *Bacillus* sp. RP242 [53]. Notably, Zhang et al. (2008) demonstrated that 100 µg/g of active compounds from *Bacillus subtilis* B-FS06 significantly inhibited mycelial growth of *A. flavus* on peanuts after 5 days of incubation, with complete inhibition achieved at 200–250 µg/g [29]. Although the concentration of our crude extract (25 mg/mL) is higher than the purified compounds used in that study, our assay extended over a much longer period (21 days vs. 5 days) and demonstrated sustained protection, with fungal contamination stabilizing after 7 days without further progression. The superior performance of the BAF143 crude extract is likely attributed to the synergistic action of multiple bioactive metabolites present in the crude preparation, as opposed to purified single compounds or fermentation broths used in previous studies. This multi-component nature likely contributes to both the high efficacy and the durable protective effect observed. Furthermore, our transcriptomic analysis revealed that the crude extract simultaneously targets cell wall integrity, induces oxidative stress, disrupts energy metabolism, and impairs stress signaling, which may collectively enhance its in vivo biocontrol performance compared to agents with single-target mechanisms.

While the present study provides comprehensive insights into the antifungal mechanism and biocontrol efficacy of the BAF143 crude extract, several limitations should be acknowledged. First, the specific chemical structures of the bioactive metabolites remain to be fully elucidated. It should be noted that the novelty of this work is not solely dependent on the discovery of new chemical entities. The key innovations include: (i) the first report of a marine-derived *Bacillus* sp. BAF143 with potently antifungal activity against *A. flavus*; (ii) the discovery of a compensatory collapse mechanism that differs fundamentally from the classic single-target or global-inhibition modes of action; and (iii) the demonstration of sustained biocontrol efficacy on peanuts with a 96.18% reduction in fungal contamination. Future studies will focus on the isolation and structural identification of the active compounds, comparison of their antifungal activities with the crude extract, and evaluation of their potential for large-scale application in post-harvest preservation.

## 4. Materials and Methods

### 4.1. Fungal Strains

The strain *Aspergillus flavus* GP15-4 [54], originally isolated from peanuts, was obtained from Prof. Bin Liu (Institute of Edible Fungi, Guangxi University). It was cultured on potato dextrose agar (PDA) at 30 °C for 5 days.

### 4.2. Preparation of Spore Suspension

*A. flavus* was cultured on PDA at 30 °C for 5 days to induce sporulation. Conidia were harvested by flooding the plate surface with sterile distilled water containing 0.1% (*v*/*v*) Tween 80. The spore suspension was gently scraped using a sterile glass spreader and filtered. The filtrate was collected and the spore concentration was determined using a hemocytometer and adjusted to 1 × 10^6^ CFU/mL with sterile distilled water.

### 4.3. Isolation of Strain BAF143

Strain BAF143 was isolated from an MT plate that had been used for the isolation of marine fungi *Nigrograna chlamydospora* B143 [55], and a single bacterial colony was picked and purified by repeated streaking on LB agar plates. The purified strain was stored in 20% (*v*/*v*) glycerol at −80 °C for long-term preservation.

### 4.4. Screening and Antifungal Activity of BAF143

The antagonistic activity of strain BAF143 against *A. flavus* was evaluated using the plate confrontation assay on three distinct media, including potato dextrose agar (PDA, a standard fungal medium), martin medium (MT, a selective fungal medium), and Luria–Bertani agar (LB, a standard bacterial medium). Briefly, wells of 5 mm in diameter were punched on each agar plate using a sterile cork borer, with one well positioned at the center and four wells evenly distributed around the periphery. A 5 µL aliquot of *A. flavus* spore suspension (1 × 10^6^ CFU/mL) was inoculated into the central well, while 5 µL of a 24 h bacterial culture of strain BAF143 was dispensed into each of the four peripheral wells. In the control group, only *A. flavus* was inoculated without bacterial treatment. All plates were incubated at 30 °C for 5 days, after which the colony diameter of *A. flavus* was measured, and the inhibition rate was calculated using the following Formula (1).

(1) Inhibition rate (%) = (d_Control_ − d_Treatment_)/d_Conrol_ × 100%

where d_Control_ and d_Treatment_ represent the colony diameters of the *A. flavus* colonies in the control and treatment groups, respectively.

### 4.5. Identification of BAF143

#### 4.5.1. Morphological Characterization

The morphological characteristics of strain BAF143 were examined on LB agar after 24 h of incubation at 30 °C. Colony morphology (size, color, shape, margin, elevation, and surface texture) was recorded according to standard microbiological methods. Gram staining was performed using a Gram stain kit (Solarbio, Beijing, China) following the manufacturer’s instructions. Cell morphology was observed with a Nikon Eclipse 80i microscope (Nikon Corporation, Tokyo, Japan) equipped with Nikon Digital Sight DSL1 microphotographic system.

#### 4.5.2. Molecular Identification and Phylogenetic Analysis

Genomic DNA of strain BAF143 was extracted from overnight cultures grown in LB broth using Chelex 100 [56]. The 16S rRNA gene was amplified by polymerase chain reaction (PCR) using the universal bacterial primers 27F (5′-AGAGTTTGATCCTGGCTCAG-3′) and 1492R (5′-GGTTACCTTGTTACGACTT-3′). The PCR products were sequenced by Beijing Liuhe BGI Gene Technology Co., Ltd. (Guangzhou, China) The sequence was compared with reference sequences available in the GenBank database using the BLASTn (v. 2.6.0, https://ncbiinsights.ncbi.nlm.nih.gov/, 10 September 2026) algorithm to identify closely related taxa. Phylogenetic analysis was performed using MEGAv6.06 [57]. The phylogenetic tree was constructed using the maximum likelihood (ML) method with 1000 bootstrap replicates to assess the robustness of the tree topology [58]. The sequence of LT630029 *Bacillus mediterraneensis* Marseille-P2366 was used as an outgroup.

### 4.6. Antifungal Activity of Crude Extracts at Different Fermentation Times

#### 4.6.1. Fermentation and Preparation of Crude Extracts

A 100 µL suspension of strain BAF143 was cultured for 24 h and inoculated into 300 mL of liquid Luria–Bertani medium in 500 mL flasks. The cultures were incubated at 30 °C on a rotary shaker at 200 rpm for 1, 2, 3, 4, 5, 6, and 7 days. At the end of each fermentation period, the entire culture broth was harvested and extracted three times with an equal volume of ethyl acetate. The combined organic phases were concentrated to dryness under reduced pressure at 40 °C using a rotary evaporator. The resulting crude extracts were weighed to determine the yield (mg/300 mL culture) and then redissolved in methanol at a stock concentration of 25 mg/mL (*w*/*v*). All extracts were stored at −20 °C in the dark until further analysis. The final concentration of methanol in all subsequent antibacterial assays was maintained below 1% (*v*/*v*) to exclude solvent interference.

#### 4.6.2. Agar Well Diffusion Assay

The agar well diffusion method was employed for preliminary screening of antibacterial activity [55]. The procedure for this assay was identical to that described in Section 4.4, with minor modifications. Briefly, three wells (5 mm in diameter) were punched on each PDA plate, one at the center and two at opposite positions on the periphery. The central well was inoculated with 5 µL of *A. flavus* spore suspension (1 × 10^6^ CFU/mL), while the two peripheral wells were each loaded with 50 µL crude extract (25 mg/mL) of strain BAF143 at varying fermentation times. Control plates received equal volumes of methanol solvent in the peripheral wells. All plates were incubated at 30 °C for 5 days, after which the colony diameter of *A. flavus* was measured, and the inhibition rate was calculated using Formula (1).

### 4.7. Poison Food Technique

According to Section 4.6.1, the bacterial fermentation liquid that had been cultured for 3 days was extracted, then concentrated and dissolved. The crude extract solvent was stored at −20 °C. The solution was filter sterilized through a 0.22 µm syringe filter before use. The final concentration of the solvent in the medium did not exceed 1% (*v*/*v*) to avoid any solvent-induced inhibition of fungal growth. PDA was prepared and autoclaved at 121 °C for 15 min. After cooling to approximately 50–55 °C, the crude extract was added to the molten PDA to achieve 10 mg/mL (as preliminary experiments confirmed that 25 mg/mL crude extract had a significant inhibitory effect, and the poison food technique requires a larger amount of crude extract, therefore, 10 mg/mL was chosen). An equal volume of methanol solvent was added to the control medium. Approximately 10 mL of the poisoned medium was poured into each sterile Petri dish (60 mm in diameter) and allowed to solidify at room temperature. Wells 5 mm in diameter were punched on each PDA plate at the center, inoculated with 5 µL of *A. flavus* spore suspension (1 × 10^6^ CFU/mL), and incubated at 30 °C for 5 days. After incubation, the colony diameter of *A. flavus* was measured and the average diameter was calculated. The inhibition rate was determined using Formula (1).

### 4.8. Minimum Inhibitory Concentration (MIC) Assay

For fermentation days that yielded observable inhibition effects in the agar diffusion assay, the minimum inhibitory concentration (MIC) was further determined using the broth microdilution method in 96-well sterile microplates. The crude extract and 1 µL *A. flavus* spore suspension (1 × 10^6^ CFU/mL) were added to each well with 100 µL PDB, resulting in a series final concentrations of 0.1953, 0.3906, 0.78125, 1.5625, 3.125, 6.25, 12.5, 25, and 50 mg/mL. Negative control (broth and spore suspension), blank control (broth only), and solvent control (methanol in broth and spore suspension) were included on each plate. The plates were incubated at 30 °C for 48 h. The MIC was defined as the lowest concentration of the extract that completely inhibited visible bacterial growth.

### 4.9. Detection of Spore Germination

For the spore germination assay, the crude extract was dissolved in PDB and dispensed in 900 µL aliquots into sterile 2 mL centrifuge tubes. Subsequently, 100 µL of *A. flavus* spore suspension (1 × 10^6^ CFU/mL) was inoculated into each tube, resulting in a final extract concentration of 25 mg/mL in a total volume of 1 mL. The negative control received an equivalent volume of methanol solvent instead of the crude extract. All tubes were incubated at 30 °C with continuous shaking at 200 rpm for 24 h. Following incubation, the number of germinated and non-germinated spores was counted under a microscope. The spore germination rate and inhibition rate were calculated according to the following Formulas (2) and (3).

(2) Spore germination rate (%) = A/B × 100%

(3) Inhibition rate of spore germination (%) = (C − D)/C × 100%

where A represents the number of germinated spores, B denotes the total number of spores counted, C is the germination rate in the control group, and D is the germination rate in the treatment group.

### 4.10. Observation of Ultrastructure

#### 4.10.1. Strain Cultivation

For SEM and TEM observation, a 10 μL spore suspension of *A. flavus* was added into a 2 mL tube with 1 mL PDB at 30 °C, 200 rpm for 24 h. After incubation, the crude extracts from BAF143 were added to the culture medium at final concentrations of MIC, 1/2 MIC and 1/4 MIC, with an equivalent volume of methanol solvent as the control group. After incubation for an additional 3 days under the same conditions, the mycelia were harvested by filtration through sterile filter paper and washed three times with 0.1 M phosphate-buffered saline (PBS, pH 7.3).

#### 4.10.2. Scanning Electron Microscopy (SEM)

The mycelial samples were harvested and processed following the method described by Lopez-llorca and Hernandez (1996) [59]. The mycelia were fixed with 2.5% glutaraldehyde at 4 °C overnight. After fixation, the samples were washed with 0.1 M phosphate-buffered saline (PBS, pH 6.8), dehydrated through a graded ethanol series (50%, 70%, 80%, 85%, 90%, 95%, and 100%, 15 min each), critically freeze-dried, and sputter-coated with gold-palladium. Observations were performed using a scanning electron microscope (FEI Quattro S, American thermoelectricity, Brno, Czech Republic).

#### 4.10.3. Transmission Electron Microscopy (TEM)

The mycelia were fixed with 2.5% glutaraldehyde at 4 °C for 12–24 h and post-fixed with 1% osmium tetroxide for 1–2 h, then were rinsed in 0.1 M PBS (pH 7.3) 3 times each for 15 min, and washed with sterile water 3 times each for 10 min. The samples were dehydrated through an ethanol series (30%, 50%, 70%, 80%, 95%, and 100%, 15 min each) and embedded in EMBed 812 at 37 °C in an oven for 48 h, then heated to 45 °C for 12 h. The samples were placed in a 60 °C oven to polymerize for more than 48 h. Ultrathin sections (60–70 nm) were cut using an ultramicrotome (Leica EM UC7, Leica Microsystems, Wetzlar, Germany) with a diamond knife. Sections were stained with uranium acetate for 10–30 min, lead citrate-stained for 5–10 min, and examined under a transmission electron microscope (JEOL 1400Flash, JEOL, Tokyo, Japan) at an accelerating voltage of 80 kV.

### 4.11. Transcriptome Analysis

Strain cultivation was performed as described in Section 4.10.1, except that only the MIC concentration was tested in the treatment group. Total RNA was extracted, the quantities were assessed, and then the libraries were constructed and sequenced on an illumina platform. Raw reads were filtered to remove adapters and low-quality reads. Clean reads were aligned to the *A. flavus* NRRL3357 reference genome (ASM901741v1) using HISAT2 [60]. Gene expression levels were quantified using featureCounts [61] and normalized as expected number of fragments per kilobase of transcript sequence per millions base pairs sequenced (FPKM) or transcripts per million (TPM) [62]. Differential expression analysis was performed using DESeq2 [63] with a threshold of |log_2_(fold change)| ≥ 1 and adjusted *p*-value (padj) < 0.05. Gene Ontology (GO) enrichment analysis was performed using Fisher’s Exact Test to identify over-represented biological processes (BPs), molecular functions (MFs), and cellular components (CCs) among the differentially expressed genes (DEGs). Kyoto Encyclopedia of Genes and Genomes (KEGG) pathway enrichment analysis was conducted to identify significantly enriched metabolic and signaling pathways [64]. Enrichment was considered statistically significant at padj < 0.05.

### 4.12. Determination of Cell Membrane Integrity

The same protocol as described in Section 4.10.1 was followed, except that the crude extracts were applied at a single concentration (the MIC value) and the incubation time was reduced to 12 h. All other parameters were kept constant. The mycelia were harvested by filtration through sterile filter paper and washed three times with 0.1 M phosphate-buffered saline (PBS, pH 7.3). The mycelial samples were incubated with 5 μg/mL propidium iodide (PI) at 37 °C for 30 min in the dark. After incubation, the samples were rinsed and resuspended in PBS buffer, and subsequently examined under a fluorescence microscope.

### 4.13. Mitochondrial Membrane Potential (MMP) Assay

The culture conditions for *A. flavus* were the same as those described in Section 4.10.1. MMP was measured using a JC-1 assay kit (Solarbio, Beijing, China) according to the manufacturer’s instructions. Fluorescence microscopic images were acquired for further analysis.

### 4.14. Superoxide Dismutase (SOD) Activity

The culture conditions for *A. flavus* were the same as those described in Section 4.10.1. The SOD activity was detected using an SOD activity assay kit (Solarbio, Beijing, China) according to the manufacturer’s instructions. Define the SOD content in the reaction system that corresponds to a 50% inhibition rate per 1 g of mycelium as one unit of SOD activity.

### 4.15. Malondialdehyde (MDA) Content Determination

The culture conditions for *A. flavus* were the same as those described in Section 4.10.1. Subsequently, the MDA content was established by using a commercial MDA assay kit (Solarbio, Beijing, China), following the manufacturer’s instructions.

### 4.16. DNA Damage

The culture conditions for *A. flavus* were the same as those described in Section 4.10.1. A stock solution of DAPI (5 mg/mL) was prepared by dissolving the dye in deionized water or dimethylformamide (DMF) and stored at −20 °C in the dark until use. For staining, the stock solution was diluted to a working concentration of 10 μg/mL using phosphate-buffered saline (PBS, 10 mM, pH 7.3). The DAPI working solution was added to completely cover the sample and incubated at room temperature for 10 min in the dark. After incubation, the mycelia were gently rinsed with PBS to remove excess dye. Fluorescence microscopic images were acquired for further analysis.

### 4.17. Biocontrol on Peanuts

Each flask was filled with 10 g of peanuts and sterilized with sodium hypochlorite and ethanol. In a detailed procedure, peanuts were soaked in 1% sodium hypochlorite for 5 min and washed with sterile water for 30 s. Subsequently, the peanuts were rinsed with 75% ethanol for 5 min and washed with sterile water. When the peanuts dried, 1 mL *A. flavus* spore suspensions (1 × 10^6^ CFU/mL) and 1 mL 25 mg/mL crude extract of BAF143 were added and then the peanuts were incubated at 30 °C for 21 days. For the control, 1 mL *A. flavus* spore suspensions (1 × 10^6^ CFU/mL) and 1 mL methanol solvent were added into the flask instead of crude extract. After incubation, the peanuts were suspended in 80 mL of sterile 0.05% (*v*/*v*) Triton X-100 solution and shaken for 10 min, after which the spore concentration was quantified using a hemocytometer.

### 4.18. Statistical Analysis

All experiments were performed in triplicate, and data are expressed as mean ± standard deviation (SD). Statistical significance was determined using one-way ANOVA followed by Tukey’s post hoc test. A *p*-value < 0.05 was considered statistically significant.

## 5. Conclusions

The strain BAF143 was identified as *Bacillus* sp. BAF143 and its crude extract exhibited strong inhibitory effects on the mycelial growth and spore germination of *A. flavus*. In summary, the crude extract from *Bacillus* sp. BAF143 exerts potent multi-pathway antifungal activity against *A. flavus* by disrupting cell wall integrity and impairing stress signaling, while triggering a paradoxical transcriptional surge in ribosome biogenesis, oxidative phosphorylation, ergosterol biosynthesis, cell cycle, DNA replication, and energy metabolism. This broad upregulation reflects a desperate compensatory attempt by the fungus to repair damage and sustain proliferation under severe stress. However, concurrent ROS accumulation, lipid peroxidation, and mitochondrial dysfunction overwhelmed these efforts, leading to membrane permeabilization, DNA fragmentation, and apoptosis-like cell death. On peanuts, the crude extract achieved a 96.18% reduction in spore count with sustained protection, positioning *Bacillus* sp. BAF143 as a promising biocontrol agent for mitigating *A. flavus* contamination and aflatoxin risks in postharvest systems.

## Figures and Tables

**Table 1:** Key differentially expressed genes involved in antifungal response.

Gene Category	Gene ID	log2FoldChange	Gene Name	Function
Cell wall synthesis	F9C07_2280790	−2.227	*CHS1*	Chitin synthase
	F9C07_3254	−3.395	*CHSC*	Chitin synthase C
	F9C07_2231477	−2.036	*CHS1*	Chitin synthase
	F9C07_2282541	−2.068	*CHSE*	Chitin synthase E
	F9C07_2233731	−2.362	*CHSG*	Chitin synthase G
	F9C07_2280269	−1.003	*FKSP*	1,3-beta-glucan synthase
Ergosterol synthesis	F9C07_2277979	1.084	*ERG* *2*	C-8 sterol isomerase
	F9C07_4097	1.794	*ERG* *4*	C-24(28) sterol reductase
	F9C07_2239	2.484	*ERG* *5*	C-22 sterol desaturase
	F9C07_4771	2.410	*ERG* *6*	C-24 methyltransferase
	F9C07_10749	1.802	*ERG* *7*	Lanosterol synthase
	F9C07_2124	1.024	*ERG* *27*	3-keto-steroid reductase
Antioxidant enzymes	F9C07_2284865	−1.701	*CatA*	Catalase A
	F9C07_2277540	2.681	*CatB*	Catalase B
	F9C07_2286715	2.346	*SOD1*	Cu,Zn superoxide dismutase 1
	F9C07_1593	1.049	*SOD2*	Cu,Zn superoxide dismutase 2
	F9C07_2717	2.71	*MnSOD*	Mn superoxide dismutase
	F9C07_2285981	−4.468	*SOD*	Superoxide dismutase
Cell cycle	F9C07_2278625	1.100	*Cdc6*	Cell division control protein
	F9C07_2277881	2.315	*Cdc14*	Cell division control protein
	F9C07_2277647	3.619	*Cdc* *20*	Cell division control protein
	F9C07_2228016	1.041	*Cdc* *45*	Cell division control protein
DNA replication	F9C07_1311959	1.078	*Mcm3*	DNA replication licensing factor
	F9C07_2062672	1.194	*Mcm7*	DNA replication licensing factor
	F9C07_4653	1.762	*RFA2*	Replication factor-a protein
	F9C07_1145905	1.209	*Dna2*	DNA replication helicase
Ribosome	F9C07_10873	2.045	*L2*	60S ribosomal protein
	F9C07_796	1.471	*L12*	60S ribosomal protein
	F9C07_2227618	1.997	*L22*	60S ribosomal protein
	F9C07_2279517	2.077	*S5*	40S ribosomal protein
	F9C07_2279774	2.061	*S11-B*	40S ribosomal protein
	F9C07_285	2.819	*S13*	40S ribosomal protein
Energy metabolism	F9C07_2226744	1.113	*pfkA*	6-phosphofructokinase
	F9C07_2286506	4.488	*GAPDH*	Glyceraldehyde 3-phosphate dehydrogenase
	F9C07_2468	1.099	*MANBA*	Beta-mannosidase
	F9C07_2446	1.616	*pgmA*	Phosphoglucomutase
	F9C07_453	2.336	*MDH2*	Palate dehydrogenase
	F9C07_2283332	1.809	*IDH3*	Isocitrate dehydrogenase (NAD+)
	F9C07_2286768	1.686	*SDHC*	Succinate dehydrogenase
	F9C07_8812	1.532	*fumC*	Fumarate hydratase
MAPK signaling pathway	F9C07_2279680	−2.057	*RLM1*	Transcription factor
	F9C07_11676	−1.165	*WSC1*	Cell wall integrity and stress response component

## Data Availability

The data presented in this study are available on request from the corresponding author.
